# Effect of ovarian growth factors on ultra-structural maturation in frozen human immature oocytes after in vitro maturation: a comparative study

**DOI:** 10.1186/s12978-022-01521-8

**Published:** 2022-12-01

**Authors:** Hakimeh Akbari, Masoud Mohammadi

**Affiliations:** Cellular and Molecular Research Center, Gerash University of Medical Sciences, Gerash, Iran

**Keywords:** Infertility, Ultrastructure, In vitro maturation, Ovarian growth factors, Oocytes

## Abstract

**Background:**

In artificial reproductive technique (ART), nearly 20% of human oocytes are immature in the germinal vesicle (GV) phase. Consequently, the best method for reserving them is cryopreserving GV oocytes, and in vitro maturation (IVM) is recommended. The aim of this study was to determine the ultrastructure characteristics of fresh and vitrified immature human oocytes after in vitro maturation in conditioned mediums.

**Methods:**

This study was a comparative laboratory study carried out in 2018 at Afzalipur Infertility Center in Kerman. 170 fresh and 198 vitrified GV oocytes were cultured within three IVM mediums; α-mem as control medium, α-mem supplemented with human bone marrow mesenchymal stem cells (BM-MSCs) and α-mem supplemented with ovarian growth factors (O.F). After 48 h, the maturation rate and morphological feature of IVM oocytes [132 fresh IVM (fIVM) and 134 vitrified IVM (vIVM)] were evaluated. For the ultrastructure study, 10 IVM oocytes from each medium were compared with 10 fresh in vivo oocytes cancelled from ART.

**Results:**

The survival rate of vitrified GV oocyte after thawing was 88.88%. The oocyte maturation rate was reduced in vIVM compared to the fIVM group (76.33% vs. 77.95%); the highest oocyte maturation rate in the O.F fIVM and lowest in α-mem vIVM (82.35% vs. 71.42%). The lowest number of cortical granules was observed in α-mem vIVM, but the greatest presence of M-SER aggregates was in O.F fIVM. In vIVM oocytes, the oolemma contained irregular little microvillus organization.

**Conclusions:**

The O.F mediums have shown the highest maturation which defends the oocyte ultra-structural conservation.

## Plain Language Summary

In artificial reproductive technique (ART), nearly 20% of human oocytes are immature in the germinal vesicle (GV) phase. Consequently, the best method for reserving them is cryopreserving GV oocytes, and in vitro maturation (IVM) is recommended. This study was a comparative laboratory study carried out in 2018 at Afzalipur Infertility Center in Kerman. 170 fresh and 198 vitrified GV oocytes were cultured within three IVM mediums; α-mem as the control medium, α-mem supplemented with human bone marrow mesenchymal stem cells (BM-MSCs), and α-mem supplemented with ovarian growth factors (O.F). After 48 h, the maturation rate and morphological features of IVM oocytes were evaluated. For the ultrastructure study, 10 IVM oocytes from each medium were compared with 10 fresh in vivo oocytes cancelled from ART. The survival rate of vitrified GV oocytes after thawing was 88.88%. The oocyte maturation rate was reduced in vIVM compared to the fIVM group (76.33% vs. 77.95%); the highest oocyte maturation rate in the O.F fIVM and lowest in α-mem vIVM (82.35% vs. 71.42%). The lowest number of cortical granules was observed in α-mem vIVM, but the greatest presence of M-SER aggregates was in O.F fIVM. In vIVM oocytes, the oolemma contained irregular little microvillus organization. The O.F mediums have shown the highest maturation with defending the oocyte ultra-structural conservation.

## Background

One effective method for preserving female fertility is oocyte cryopreservation in a clinical trial [1]. On the other hand, ethical issues can lead to avoiding embryo cryopreservation; accordingly, oocyte cryopreservation is a critical technique for returning fertility and offering oocyte banks to maintain women’s fertility [2].

In the ovarian stimulation during assisted reproductive technology (ART), approximately 20% of human oocytes are immature; thus, cryopreserving immature oocytes in the germinal vesicle (GV) stage and then in vitro maturation (IVM) can suggest compensations; moreover, the use of immature oocytes for cryopreservation may circumvent some of the limitations associated with the vitrification and thawing of mature oocytes, specifically relating to the functional integrity of the meiotic spindle and policy of resulting embryos [3–5]. However, a low survival rate of immature oocyte cryopreservation was reported, which is a serious problem [6].

Nevertheless, vitrified MII oocytes show poor developmental competence [7]. While the GV stage oocytes are as vulnerable to cryoinjuries as the mature stage oocytes, some recovery mechanisms may occur during culturing in the IVM medium; however, one of the disadvantages of GV oocyte cryopreservation is that the vitrified GV oocyte must be thawing before IVM [8, 9].

Some factors, such as the oocyte meiotic stages and the warming and cooling rates of vitrified oocytes, influence the cell’s capacity to survive cryopreservation [10]. The disruption of cellular structure and plasma membrane and de-polymerization of microfilaments or microtubules are common complications of cryopreservation; these adverse side effects depend on the kind of cryopreservation protocols, the developmental stage and maturation mediums are changed [10, 11].

There are no general IVM protocols; in order to reach better outcomes, numerous alterations, such as cycle monitoring and purifying culture conditions, have been recommended [12]. Mesenchyme stem cells (MSCs) originate in various tissues, including bone marrow and placental tissue, which can be used for potential progressive therapy [13].

In vitro studies have shown that bone marrow stem cells actively prevent the function of several immune cells through secreted growth factors, cytokines and enzymes; similarly, ovarian growth factors mediums might provide beneficial elements to the culture medium, counting vitamins, cytokines and amino acids, which affect serum component that useful for oocyte nuclear maturation and cytoplasmic development [14–16].

Several studies showed an association between the rate of fertilization with IVM oocyte morphological quality. Nevertheless, phase contrast microscopy could not reveal analytical signs of oocyte quality and cytoplasmic developmental capability. In contrast, electron microscopy can be an effective device for assessing the ultrastructure features of IVM oocytes [17, 18]. The aim was to determine the ultrastructure characteristics of fresh and vitrified immature human oocytes after in vitro maturation in conditioned mediums.

## Methods

### Study period and patients

This study was a comparative laboratory study carried out in 2018 at Afzalipur Infertility Center in Kerman. A total of 368 germinal vesicles (GV) oocytes were obtained from 184 patients (18–46 years old) who practised an ICSI cycle. All the patients underwent evaluation by the Kerman Medical University’s Ethical Committee and were admitted to Afzalipour Hospital of Kerman, Iran.

### Ovarian stimulation

Ovarian stimulation was attained by long protocol via administration of a combination of (GnRH) gonadotropin-releasing hormone and follicular stimulating hormone (FSH). Following follicular growth via transvaginal ultrasound, when adequate matured follicle was reached, injection of human chorionic gonadotropin (hCG) (Switzerland, IBSA Co) 10,000 IU was started; 36 h later, the oocyte collection was carried out through laparoscopy [19].

### Oocyte preparation

Cumulus oocyte complexes (COCs) were picked up and transferred in G-IVF (Vitrolife, Sweden) culture medium under mineral oil (Vitrolife, Sweden) for 2–3 h in an incubator. The COCs were denuded via mechanical pipetting dissection using 80 IU hyaluronidase (Sigma Co, USA). Nuclear maturity of the denuded oocytes was assessed under the dissecting microscope, according to that be the presence of the first Polar Body (PB); accordingly, oocytes were classified as immature (GV or MΙ) or mature (MII). MII oocytes were used for IVF or ICSI procedures and GV oocytes, vitrified or fresh, were cultured in vitro [8].

Germinal vesicle oocytes were studied in six groups after being denuded. For the first two groups, 1: fresh GV oocytes were matured in three vitro maturation mediums called (fIVM). 2: GV oocytes were vitrified and then maturated after thawing (vIVM). Both of them were maturated in three vitro maturation mediums.

### IVM mediums

#### Three types of IVM culture mediums were used in this study

- (Medium 1) as the based control medium: is Alpha Minimum Essential Medium (α-mem) [20].
- (Medium 2) α-mem + supernatants derived from human bone marrow mesenchyme stem cells (B.M) α-mem + supernatants derived from human bone marrow mesenchyme[21].
- (Medium 3) α-mem + ovarian factor (O.F) including: [3 ng/ml of BDNF, 100 ng/ml of IGF-I, 1 mg/ml of estradiol, 30 ng/ml of GDNF, 10 ng/ml of leptin, 0.5 ng/ml of FGF2] [15].

### Vitrification

The germinal vesicle oocytes were put in the first medium containing Ham’s F10 medium + 20% human serum albumin (HSA) (Plasbumin Co, USA). Next, they were transferred in an equilibration solution (ES) containing 7.5% dimethyl sulphoxide (DMSO) with 7.5% ethylene glycol (EG) at room temperature for 10 min. Finally, these oocytes were removed to the vitrification solution (VS) containing 0.5 mol/l sucrose with 15% DMSO and 15% EG for 50–60 s at room temperature. Then the vitrified oocytes were placed on the cryotops (Vitrolife, Sweden) and placed into the fluid nitrogen storage tank instantly for reserving several months [5].

### Thawing

The thawing process of the vitrified oocytes was done in the thawing solution [Ham’s F10 medium containing 20% (HSA) as a based medium] through locating these oocytes in different mediums in four steps: first step: 1.0 mol/l sucrose for 50–60 s, second step: 0.5 mol/l sucrose for 3 min, the third step: 0.25 mol/l sucrose for 3 min and finally these oocytes were located in Ham’s F10 medium with 20% (HAS) for 3–5 min. Consequently, the oocytes were placed randomly in one of the three IVM mediums for 48 h in an incubator. Then the oocyte viability was assessed by stereomicroscope [4, 22].

### MSC isolation and culture

Human bone marrow mesenchymal stem cells were provided from Afzalipour Kerman Medical University (Kerman-Iran) (α-mem) as the based control medium. These cells were cultured and Vitrified according to Ling’s method [23]; human bone marrow mesenchymal stem cells were washed with phosphate buffered saline (PBS) containing 100 mg/ml streptomycin (Gibco), 100 U/ml penicillin (Gibco) and collagenase I (Sigma), then, these cells cultured in α-mem medium (α-mem, Gibco) supplemented with 10% FBS (Gibco), 100 mg/ml streptomycin (Gibco) and 100 U/ml penicillin (Gibco), in the incubator. The medium was changed, and they were trypsinised after attaining complete cell confluency. After changing the medium and gathering supernatant, it was filtered with a 0.2 µm membrane for immediate use [8].

### Electron microscopy

To examine the oocyte ultrastructure by transmission electron microscopy (TEM), 10 numbers IVM matured oocytes (MII) from each medium were compared with in-vivo MII oocytes from patients that were cancelled ICSI. For the TEM study, the oocytes were prepared according to Nottola et al. method [17]. The oocytes were fixed in a solution of glutaraldehyde 1.5% (Sigma, USA) within 0.1 M phosphate-buffered saline (PBS) for 2–5 days at 4 °C; these oocytes were washed by PBS buffer for 10 min; next, the oocytes were fixed in a solution of osmium tetroxide 1% (Agar, UK) within PBS buffer away from the light, then the oocytes washed again in PBS buffer. They were put in small thin blocks of agar 1% (Sigma, USA) for facilitating oocyte removal. The oocytes were dehydrated in rising ethanol concentrations, immersed in propylene oxide for solvent replacement and completely embedded in Epon 812 resin (Agar, UK). Semi-thin sections (0.5–1 μm thickness) were stained with toluidine blue for light microscopy evaluation (Zeiss, Germany). Ultrathin sections (60–80 nm thickness) were provided, and then uranyl acetate (7 min) and lead citrate (13 min) were stained. Finally, these sections were photographed at 80 kV by TEM (Zeiss, Germany) [9]. The oolemma integrity, zona pellucida (ZP), perivitelline space (PVS), quality of the cytoplasmic organelles and presence of polar body were assessed by light microscopy and TEM [9].

### Statistical analysis

Differences in the ultra-structural parameters in matured oocytes between the groups were compared using the χ^2^ test in SPSS software (Version 21, USA). A p-value of < 0.05 was considered statistically significant.

## Results

After thawing, the viability of vitrified oocytes was evaluated. 22 Vitrified GV oocytes were degenerated out of a total of 198 Vitrified GV oocytes, therefore 176 alive thawed oocytes entered to maturation media (Table 1).

**Table 1:** The survival rate of vitrified GV oocyte after thawing

Variable	Fresh GV oocyte	Vitrified GV oocyte
Total GV oocyte (n)	170	198
Degenerated oocyte after thawing (n)	–	22
Survival rate after thawing (%)	–	88.8%
Total IVM oocyte	170	176

### Oocyte maturation after 24 h in IVM medium

In medium1 (α-mem): Oocyte maturation rates were reduced in vIVM (71.4%), compared to fIVM (72.5%), while in medium2 (α-mem + supernatants derived from human bone marrow mesenchymal stem cells), rates of oocyte maturation in vIVM (77.9%), in comparison with, fIVM (78.9%) and maturation rate in ovarian growth factor (O.F) mediums were significantly higher than those of mediums (82.3%) (p < 0.05) (Table 2).

**Table 2:** The maturation rate and nuclear maturation stage of oocytes in groups

Groups of IVM	Number of IVM GV oocyte	Arrest in GV stage	Arrest in GVBD stage(n)	Oocyte maturation rate (%)
fIVM in α-mem	62	7	10	72.5
vIVM in α-mem	63	15	3	71.4
fIVM in B.M	57	9	3	78.9
vIVM in B.M	59	7	6	77.9
fIVM in O. F	51	3	6	82.3
vIVM in O. F	54	5	6	79.6
p value	0.503	0.418	0.221	0.000*

*p < 0.05

### Ultrastructure of MII oocyte

The oolemma of control MII oocytes which were continuous and contained several long thin microvilli, were regularly dispersed on this oolema, except in the region of polar body extrusion.

**Fig. 1:**
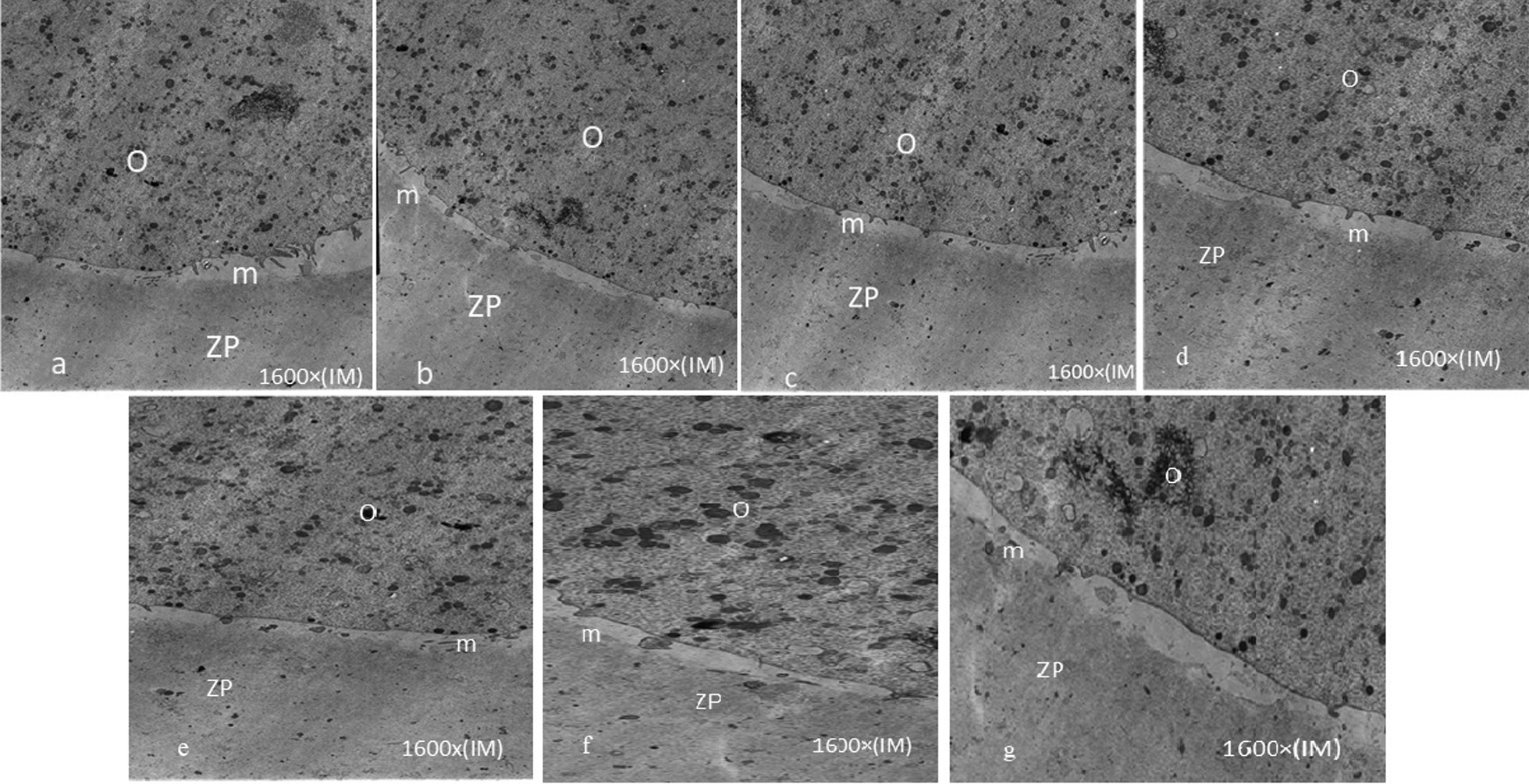
General fine structure and organelle microtopography are shown by transmission electron microscopy. Control oocyte (**a**), fIVM α-MEM (**b**), vIVM α-MEM (**c**), fIVM B.M (**d**), Vıvm B.M (**e**), fIVM O.F (**f**), vIVM O.F (**g**). Transmission electron microscopy (TEM) shows the general morphology and organelle microtopography. *O* oocyte; *ZP* zonapellucida; *m* microvilli

The zona pellucida (ZP) was composed of a thoroughly packed electron-dense fibrilar substantial (Fig. 1). The perivitelline space (PVS) was constant, with occasional debris (Fig. 1). Round cortical granules with an electron-dense arrival were located directly underneath the oolemma. The widespread of the oocyte organelles involved aggregates of smooth endoplasmic reticulum (SER) enclosed by round or oval-shaped mitochondria (M-SER aggregates) (Fig. 1).

### Ultrastructure of M-II oocyte in both mediums

**Fig. 2:**
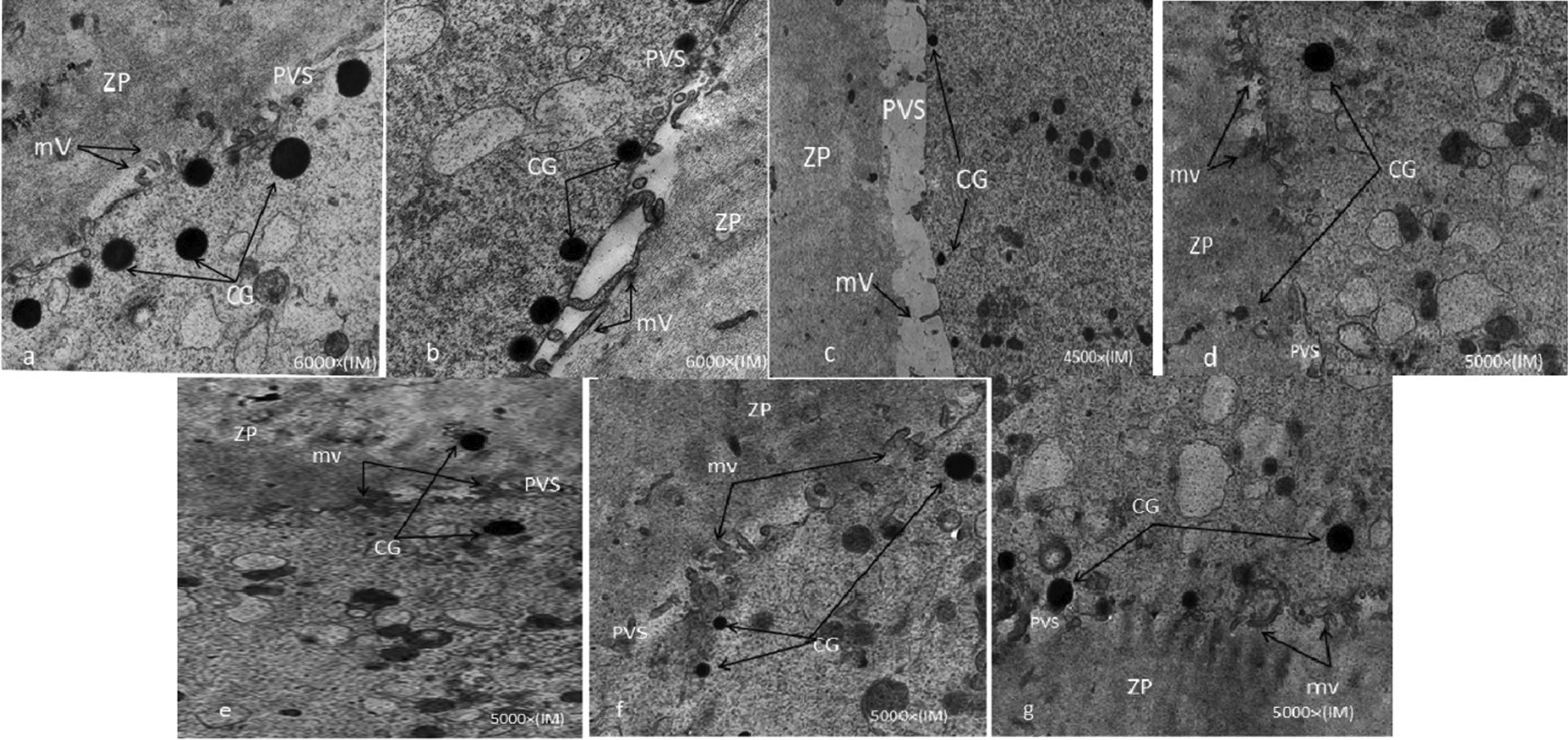
Ultrastructure of control oocyte (**a**), fIVM α-MEM (**b**), vIVM α-MEM (**c**), fIVM B.M (**d**), Vıvm B.M (**e**), fIVM O.F (**f**), vIVM O.F (**g**). Round cortical granules with an electron-dense arrival located just underneath the oolemma. The number of cortical granules was reduced in vIVM oocytes rather than f IVM. Note the increased compaction of the inner aspect of the ZP in vIVM groups. Several long Microvilli are seen in the control oocytes (**a**) and fIVM O.F (**f**). Also, long microvilli were dispersed on the oolemma of fresh IVM oocytes (**b**, **d**, **f**) rather than in vitrified IVM oocytes (**c**, **e**, **g**), while in the vitrified IVM oocytes, the oolemma was determined to have irregular and little microvillous arrangements. *ZP* zonapellucida; *mv* microvilli; *CG* cortical granules; *PVS* perivitelline space

This study noticed that the number of cortical granules was reduced in vIVM oocytes compared to fIVM, as fewer cortical granules occur in vIVM in mem medium. Also, the greatest presence of M-SER aggregates in fIVM was in the ovarian factor culture medium (Fig. 2).

### Ultrastructure of MII oocyte in the fIVM and vIVM group

Assessment of semi-thin sections under the light microscope often exposes structural impairments unnoticeable by stereomicroscope that showed minor differences between vitrified and fresh IVM oocytes, such as irregular shape and larger perivitelline space within vIVM groups. No significant differences in size, shape and total organelle distribution were found between fresh and vitrified oocytes. In vIVM oocytes, the cortical granules presence was less than in fresh oocytes (Fig. 2). The zonapellucida thickness of the vitrified oocytes was increased. The oolemma was unbroken and continuous in the IVM oocytes inside both mediums detected. The oolemma had some long microvilli in fresh IVM oocytes, while in vitrified IVM oocytes, the little microvillous was irregular (Fig. 2).

**Fig. 3:**
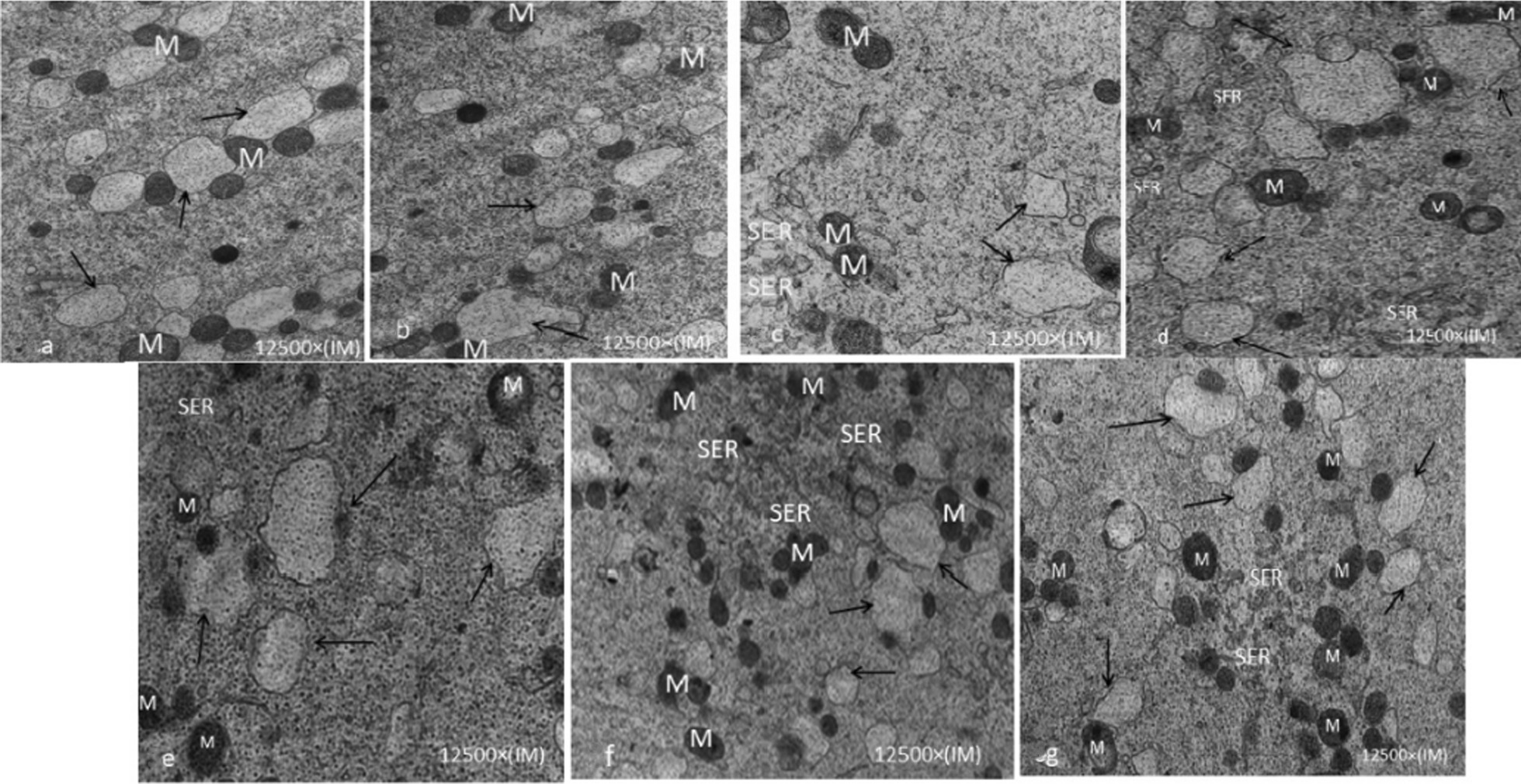
Control oocyte (**a**), fIVM α-MEM (**b**), vIVM α-MEM (**c**), fIVM B.M (**d**), Vıvm B.M (**e**), fIVM O.F (**f**), vIVM O.F (**g**). Mitochondria are generally rounded and provided with few peripheral or transverse cristae. Note the presence of complexes between mitochondria and vesicles of SER (arrows). The MV complexes were increased in IVM oocytes; Mitochondria-smooth endoplasmic reticulum (M-SER aggregates) varied in size and shape. *SER* smooth endoplasmic reticulum; *M* mitochondria; *M-SER* Mitochondria-smooth endoplasmic reticulum aggregates

Mitochondria-smooth endoplasmic reticulum (M-SER aggregates) were different in shape and size between fIVM and vIVM groups; consequently, in vIVM groups, the mitochondria were oval or slender in shape and smaller in size than fIVM (Fig. 3).

## Discussion

Cryopreservation of immature human oocytes and in vitro maturation (IVM) is the best choice for conservative IVF treatment. The advantages of IVM are avoiding side effects of ovarian hyperstimulation drugs, ovarian hyperstimulation syndrome (OHSS) and simple protocol that reduces infertility treatment costs [6, 24]. In this study, the maturation rate of oocytes was reduced in the vIVM compared to the fIVM group (76.33% vs77.95%), with the highest maturation in O.F fIVM oocytes and the lowest in α-mem vIVM (82.35% vs71.42%); accordingly, maturation rate in ovarian growth factors mediums was higher than α-mem medium in fresh and vitrified groups. Ling et al. indicated that conditioned medium (CM) of Mesenchymal stem cells (MSCs) produced a higher oocyte maturation rate (91.2%) than α-MEM (63.5%) in mice [23]. Their results differ from our outcomes, which may be due to differences in the method of culture of CM, source of Mesenchymal stem cells and oocytes species. Also, Shahidi et al. described that the maturation rates of their human GV oocytes were higher in fIVM than vIVM (75.33% vs. 45.92%) [18], which are in agreement with our findings, nevertheless our maturity rates following IVM were higher, which is maybe due to variances in the IVM mediums. Recent studies showed that cryotop vitrification is a prospective cryopreservation technique which prominently improves the maturation and good survival rate of oocytes; our current data is also consistent with that of Fasano et al. [3].

### Ultrastructure of M-II oocyte in both mediums

Bone marrow mediums improved the rate of in vitro oocyte maturation and oocyte viability after thawing better than α-MEM. On the other hand, Parekkadan et al. showed that almost 30% of MSC-CM consisted of an enormous variety of molecules involved in immunomodulation and chemotactic cytokines and growth factors as potential mediators of the therapeutic effect of Mesenchyme condition medium [21].

### Ultrastructure of M-II oocyte in fIVM and vIVM group

The results indicated that the maturity was greater in fIVM rather than vIVM. The vitrified IVM oocytes seemed regular shape and ooplasm uniformity under light microscopy examination, as in the fresh IVM oocytes in both groups. According to some studies in agreement with ours, good conservation of vitrified oocytes and conservation protocols does not significantly damage oocyte features [17, 25, 26]. On the other hand, Boonkusol et al. noticed that the vitrification process affects ultra-structural conditions of the mature oocyte [27]. Their outcomes are different from ours, which may be due to differences in the source of oocyte and oocyte meiosis stage, which were vitrified and similar to our findings.

Moreover, the microtubular spindle damage during cryopreservation in human GV oocytes is less than in mature oocytes [9, 22], which could indicate the reason for the high survival and maturation rate in vitrified GV oocytes in this study. In vIVM oocytes, the presence of cortical granules is less than that in fIVM. Several studies support our results for ZP hardening and a decrease in the number of cortical granules after vitrification, similar to other findings [4, 28–30]. This study detected that both fresh and vitrified IVM oocytes were bounded by continuous oolemma. This finding was in agreement with Khalili et al. and Notolla et al. [5, 17].

On the other hand, microfilaments are involved in fertilization development and cleavage rate, which can be improved by cry protectants and cooling [11]. Rojas et al. detected that the variation in spindle microtubules and absent microfilaments occurred after vitrification [10]. In this study, in fIVM oocytes some long microvilli were dispersed on the oolemma rather than vIVM oocytes determined to have irregular and little microvillus arrangements; this was in agreement with Notolla et al. [17]. After cryopreservation, oocytes must recover the cytoskeleton damage that might affect cell division and survival [30]. Swain et al. showed that the cause of fewer fertilization rates and developmental potency of cryopreserved oocytes might be unsuitable microvilli distribution [31]. Shahedi et al. described that in vitrified IVM oocytes, the differences in M-SER aggregates are probably due to ethylene glycol (EG) in the vitrification process, not the cryo injury during vitrification [26]. In fIVM and vIVM oocytes, the mitochondria structure and mitochondria–vesicle complexes were similar in agreement with Nottola et al. results [17]. The variations in the M-SER aggregates organization and Mitochondria may lead to a disorder in calcium homeostasis that influences reproduction outcomes by controlling the offers of calcium concentrations and adenosine triphosphate (ATP) manufacture [32, 33].

On the other hand, Jones et al. noticed that within cryopreserved human oocytes, mitochondria couldn’t form normal aggregates and ATP production; but may have a reduced capacity to control intracellular free calcium levels [34]. According to this study, these proper mitochondrial structural may reflect that human oocytes could be more tolerant to vitrification; similarly, Izadi et al. showed that in mouse oocytes, too, mitochondria maintain good tolerance against vitrification [9]. Also, differences in M-SER aggregate sizes and shapes showed marks of injury in SER tubules and mitochondria, which could adversely affect fertilization and developmental competence [35].

### Limitations

The most important limitation of this study was the lack of access to the larger sample volume used in this study and the loss of 22 vitrified GV oocytes.

## Conclusion

The vitrification and IVM of human GV oocytes are safe methods for fertility preservation by protecting the cytoplasmic ultrastructure. The ovarian factors IVM mediums showed better maturation and cytoplasmic development by oocyte protection from any cryo-damage than other mediums. The proposed may be suitable candidate media for oocyte culture in IVF.

## Data Availability

Datasets are available through the corresponding author upon reasonable request.

## Abbreviations

ART: Artificial reproductive technique
GV: Germinal vesicle
IVM: In vitro maturation
OF: Ovarian growth factors
MSCs: Mesenchyme stem cells

## References

[1] Argyle CE, Harper JC, Davies MC (2016). Oocyte cryopreservation: where are we now?. Hum Reprod Update.

[2] Quaas AM, Pennings G (2018). The current status of oocyte banks: domestic and international perspectives. Fertil Steril.

[3] Fasano G (2010). Cryopreservation of human failed maturation oocytes shows that vitrification gives superior outcomes to slow cooling. Cryobiology.

[4] Akbari H (2020). The effect of conditioned media on mouse oocytes ultrastructure following in vitro maturation. Gene Rep.

[5] Khalili MA (2017). Vitrification of human immature oocytes before and after in vitro maturation: a review. J Assist Reprod Genet.

[6] Fernández-Reyez F (2012). Viability, maturation and embryo development in vitro of immature porcine and ovine oocytes vitrified in different devices. Cryobiology.

[7] Fujihira T, Nagai H, Fukui Y (2005). Relationship between equilibration times and the presence of cumulus cells, and effect of taxol treatment for vitrification of in vitro matured porcine oocytes. Cryobiology.

[8] Akbari H (2017). Mesenchymal stem cell-conditioned medium modulates apoptotic and stress-related gene expression, ameliorates maturation and allows for the development of immature human oocytes after artificial activation. Genes.

[9] Izadi M (2018). Assessment of mouse oocytes ultrastructure following vitrification before and after in vitro maturation. Int J Morphol.

[10] Rojas C (2004). Vitrification of immature and in vitro matured pig oocytes: study of distribution of chromosomes, microtubules, and actin microfilaments. Cryobiology.

[11] Akbari H (2018). conditioned mediums and human oocytes in vitro maturation. Int J Pharm Phytopharmacol Res.

[12] Nogueira D, Sadeu JC, Montagut J. In vitro oocyte maturation: current status. In: Seminars in reproductive medicine. Thieme Medical Publishers; 2012. 10.1055/s-0032-1311522 22585631

[13] Laroye C (2019). Bone marrow vs Wharton’s jelly mesenchymal stem cells in experimental sepsis: a comparative study. Stem Cell Res Ther.

[14] Minonzio G (2014). Frozen adipose-derived mesenchymal stem cells maintain high capability to grow and differentiate. Cryobiology.

[15] McElroy SL (2010). Parthenogenic blastocysts derived from cumulus-free in vitro matured human oocytes. PLoS ONE.

[16] Del Collado M (2016). Influence of bovine serum albumin and fetal bovine serum supplementation during in vitro maturation on lipid and mitochondrial behaviour in oocytes and lipid accumulation in bovine embryos. Reprod Fertil Dev.

[17] Nottola SA (2008). Ultrastructure of human mature oocytes after slow cooling cryopreservation with ethylene glycol. Reprod Biomed Online.

[18] Shahedi A (2013). The effect of vitrification on ultrastructure of human in vitro matured germinal vesicle oocytes. Eur J Obstet Gynecol Reprod Biol.

[19] Boots C (2016). Ovarian stimulation in the luteal phase: systematic review and meta-analysis. J Assist Reprod Genet.

[20] Mota GB (2015). Insulin influences developmental competence of bovine oocytes cultured in α-MEM plus follicle-simulating hormone. Zygote.

[21] Parekkadan B (2007). Mesenchymal stem cell-derived molecules reverse fulminant hepatic failure. PLoS ONE.

[22] Akbari H (2017). The effect of conditioned media on human oocyte maturation and developmental competence. Pharmacophore.

[23] Ling B (2008). Effect of conditioned medium of mesenchymal stem cells on the in vitro maturation and subsequent development of mouse oocyte. Braz J Med Biol Res.

[24] Jahromi BN (2018). Ovarian hyperstimulation syndrome: a narrative review of its pathophysiology, risk factors, prevention, classification, and management. Iran J Med Sci.

[25] Sun L (2017). Exosomes derived from human umbilical cord mesenchymal stem cells protect against cisplatin-induced ovarian granulosa cell stress and apoptosis in vitro. Sci Rep.

[26] Shahedi A (2013). Ultrastructure of in vitro matured human oocytes. Iran Red Crescent Med J.

[27] Boonkusol D (2007). Effects of vitrification procedures on subsequent development and ultrastructure of in vitro-matured swamp buffalo (*Bubalus bubalis*) oocytes. Reprod Fertil Dev.

[28] Khalili MA (2012). Ultrastructure of human mature oocytes after vitrification. Eur J Histochem.

[29] Chatterjee A (2017). Effects of cryopreservation on the epigenetic profile of cells. Cryobiology.

[30] Rienzi L (2017). Oocyte, embryo and blastocyst cryopreservation in ART: systematic review and meta-analysis comparing slow-freezing versus vitrification to produce evidence for the development of global guidance. Hum Reprod Update.

[31] Swain JE, Pool TB (2008). ART failure: oocyte contributions to unsuccessful fertilization. Hum Reprod Update.

[32] Nottola SA (2016). Freeze/thaw stress induces organelle remodeling and membrane recycling in cryopreserved human mature oocytes. J Assist Reprod Genet.

[33] Coticchio G (2016). Ultrastructure of human oocytes after in vitro maturation. MHR Basic Sci Reprod Med.

[34] Jones A (2004). Cryopreservation of metaphase II human oocytes effects mitochondrial membrane potential: implications for developmental competence. Hum Reprod.

[35] Eichenlaub-Ritter U (2011). Age related changes in mitochondrial function and new approaches to study redox regulation in mammalian oocytes in response to age or maturation conditions. Mitochondrion.

